# Enhanced recovery after surgery (ERAS) protocols for colorectal cancer in Japan

**DOI:** 10.1186/s12893-015-0079-0

**Published:** 2015-07-28

**Authors:** Dai Shida, Kyoko Tagawa, Kentaro Inada, Keiichi Nasu, Yasuji Seyama, Tsuyoshi Maeshiro, Sachio Miyamoto, Satoru Inoue, Nobutaka Umekita

**Affiliations:** Colorectal Surgery Division, National Cancer Center Hospital, 5-1-1 Tsukiji, Chuo-ku, Tokyo, 1040045 Japan; Department of Anesthesiology, Tokyo Metropolitan Bokutoh Hospital, 4-23-15 Koto-bashi, Sumida-ku, Tokyo, 1308575 Japan; Department of Surgery, Tokyo Metropolitan Bokutoh Hospital, 4-23-15 Koto-bashi, Sumida-ku, Tokyo, 1308575 Japan

**Keywords:** ERAS, Colorectal cancer, Japan, Length of hospital stay, Fast-track surgery

## Abstract

**Background:**

Japan has one of the highest five-year relative survival rates for colorectal cancer in the world, with its own traditions of perioperative care and a unique insurance system. The benefits of enhanced recovery after surgery (ERAS) protocols in the Japanese population have yet to be clarified.

**Methods:**

We evaluated 352 consecutive cases of colorectal cancer resection at Tokyo Metropolitan Bokutoh Hospital between July 2009 and November 2012. Of these, 95 cases were performed according to traditional protocols (traditional group), and 257 according to ERAS protocols (ERAS group), which were introduced to the hospital in July 2010. Primary endpoints included length of postoperative hospital stay, postoperative short-term morbidity, and rate of readmission within 30 days. Intensive pre-admission counselling, no pre- and postoperative fasting (provision of oral nutrition), avoidance of sodium/fluid overload, intraoperative warm-air body heating, enforced postoperative mobilization, and multimodal team care were among the main changes brought about by the introduction of ERAS protocols.

**Results:**

The median (interquartile range) length of postoperative hospital stay was 10 (10–12.75) days in the traditional group and seven (6–8) days in the ERAS group, i.e., a three-day reduction (p < 0.05) in the ERAS group. Moreover, the proportion of patients discharged within one week dramatically increased from 1 % to 77 % in the ERAS group. The overall incidence of grade 2 and 3 postoperative complications according to the Clavien-Dindo classification was 9.5 % in the traditional group and 9.3 % in the ERAS group, and 30-day readmission rates were 8.3 % and 6.6 % in the traditional and ERAS groups, respectively. There were no significant differences between the two groups. Although operative time and blood loss did not differ significantly between the two groups, the volume of intraoperative infusion was significantly decreased in the ERAS group (p < 0.05), possibly due to ERAS recommendations to avoid dehydration (i.e., avoidance of sodium/fluid overload, no preoperative fasting).

**Conclusion:**

ERAS protocols for colorectal surgery helped reduce the length of postoperative hospital stay without adversely affecting morbidity, indicating that ERAS protocols are feasible and effective in Japanese settings as well.

## Background

Enhanced recovery after surgery (ERAS), first introduced in 2005 [1], is a combination of various perioperative patient care methods based on a multimodal approach that integrates evidence-based interventions to reduce surgical stress, maintain postoperative physiological function, and accelerate recovery in patients undergoing major surgery. The fundamental components of ERAS include patient education, no fasting, optimal fluid management, decreased tube use, opioid-sparing analgesia, and early mobilization [1]. ERAS Society recommendations regarding perioperative care in colorectal surgery are continuously updated as new information becomes available [2–4].

ERAS protocols are aimed primarily at achieving early recovery, which leads to a shorter hospital stay without adversely affecting morbidity. Many studies have evaluated ERAS with a particular focus on changes in the length of hospital stay (LOS). Several meta-analyses of randomized trials in colorectal surgery showed a decrease in LOS with ERAS, compared with traditional care, without compromising patient safety [5]. However, LOS as a surrogate measure of recovery has some issues, as it is influenced by a number of non-clinical factors that differ by country, including cultural and traditional background and insurance status. It should also be noted that previous reports on the outcomes of ERAS have been largely limited to European countries and the United States [6, 7], with few studies conducted in Japan and other Asian countries [8]. In fact, some elements of ERAS do not always fit well in the Japanese setting [9].

Japan has one of the highest five-year relative survival rates for colorectal cancer in the world [10], which reflects high-quality perioperative care and surgical techniques. In Japan, D3 lymph node dissection is performed as a standard procedure for advanced colorectal cancer [11]; this is not the case in Western countries. For lower rectal cancer, the standard treatment in Asia is surgery including lateral lymph node dissection (LLND) [12], whereas preoperative chemoradiotherapy followed by surgery without LLND is the common approach in Western countries. Also, perioperative care differs between Japan and Western countries [13] (differences also exist among European countries). For example, traditional perioperative care in Japan includes *nil per os* for several days.

Japan also has a unique public health insurance system, i.e., universal health care. As this national health insurance system covers 70-90 % of all medical costs, patients in Japan need not pay much for hospital admission. Consequently, early discharge is not a priority, and some patients are reluctant to accept shorter hospital stays. The median LOS after colorectal cancer surgery was 10–19 days in 2007 and 2008 [14].

ERAS protocols were introduced to Japan at around 2010 and have since spread steadily to clinical institutions across the country [9]. However, it remains unclear whether ERAS leads to a shorter LOS with safety equivalent to that of traditional care in Japanese general hospitals, given the country’s traditions and unique health insurance system.

Recently, the utility and feasibility of ERAS for gastric surgery have been reported in Japan [15, 16]. However, to the best of our knowledge, no English report on the use of ERAS protocols for colorectal surgery in Japan exists. Therefore, this study aimed to evaluate the efficiency and safety of ERAS protocols for colorectal cancer in Japan.

## Methods

### Study population

A total of 352 consecutive patients undergoing resections for primary colorectal cancer between July 2009 and November 2012 at Tokyo Metropolitan Bokutoh Hospital, a standard Japanese general hospital, were included in this study. Exclusion criteria were emergency operations due to bowel obstruction caused by colorectal cancer (n = 38), bowel perforation with peritonitis (n = 12), and conversion surgery after long preoperative chemotherapy (n = 2). Patients who underwent stoma construction (n = 31) were also excluded, because they usually require more than two weeks before discharge in order to acquire sufficient stoma care skills. During the first year of the study, patients were treated according to care routines considered traditional at that time in Japan (traditional group, n = 95). Since specific ERAS protocols were introduced in July 2010, consecutive patients treated after 2010 were classified as the ERAS group (n = 257). Thereafter, ERAS protocols have been adopted as standard protocols for all patients undergoing colorectal resection at our institution. Throughout the study period, the same colorectal staff surgeon primarily cared for all patients, and the same team of surgeons performed all procedures. The technical aspects of surgery, such as the choice of staplers and other instruments, as well as the antibiotics used, did not differ during the study period. This study was approved by the Institutional Review Board (IRB) of Tokyo Bokutoh Metropolitan Hospital (IRB code: 25 –Heisei23).

### Perioperative protocols

The main differences between ERAS protocols adopted by Tokyo Metropolitan Bokutoh Hospital and traditional care practices (Table 1) include intensive pre-admission counseling (by both surgeons and anesthesiologists), no pre- and postoperative fasting (provision of oral nutrition), no nasogastric tube use after operation, avoidance of sodium/fluid overload, short incisions, intraoperative warm-air body heating, enforcement of postoperative mobilization, stimulation of gut motility (use of oral magnesium oxide), early urinary catheter removal, and multimodal team care. Some elements of ERAS, such as the use of thoracic epidural anesthesia/analgesia and avoidance of pre-anesthetic medication, had already been practiced routinely as part of traditional perioperative care at the time the study was initiated. The same discharge criteria (e.g., ability to tolerate solid food, adequate pain control, independence in basic activities of daily living, patient consent to discharge) were used throughout the study period.

**Table 1:** Changes in perioperative care

	Traditional care	ERAS
Preoperative counseling	only by surgeons	intensive (by both surgeons and anesthesiologists)
Preoperative fasting (oral intake)	no food on the previous day	normal diet until the previous evening
	no drink after the previous noon	drink oral hydration solution (OS-1^R^) until 3 hours before surgery*
Preoperative bowel preparation	usually	sometimes for colon cancer, and always for rectal cancer
Perioperative fluid management (avoidance of sodium/fluid overload)	no	yes (goal-directed fluid therapy)
Short incisions/lapascopic surgery	no	always
Intraoperative warm-air body heating	sometimes	always
Nasogastric tube	used (remove at POD1)	not used
Postoperative fasting	no oral intake for 3 days postoperatively	initiate oral hydration (OS-1^R^) on the morning of POD1*
	start eating soup at POD5	start eating rice at POD3
Routine postoperative mobilization care	yes (walk by POD2)	enforced (walk in the morning of POD1)
Non-opiate oral analgesics/NSAIDs	no	given routinely
Stimulation of gut motility	no	yes (use of oral magnesium oxide)
Early urinary catheter removal	no	yes
Multimodal approach	few cases	every case
Anesthesia and analgesics	combination of epidural analgesia and general anesthesis (use of remifentanil)	
Avoidance of pre-anesthetic medication (no pre-medication)	Yes	
Abstinence from smoking and drinking	Yes	

*Three 500-ml plastic bottles of oral rehydration solution [OS-1^R^; Otsuka Pharmaceutical, Tokushima, Japan]

### Data collection

Patient demographic and perioperative data, including sex, age, body mass index, American Society of Anesthesiologists (ASA) performance status, tumor location, surgical approach, colorectal cancer stage, operative time, blood loss, volume of intraoperative infusion, postoperative LOS, and complications, were collected. Complications occurring within 30 days postoperatively were defined as grade 2 or higher according to the Clavien-Dindo classification. The number of dissected lymph nodes, as confirmed by pathologists, was also measured as an indicator of the quality of cancer surgery.

### Statistical analysis

Demographic and perioperative data are presented as medians (interquartile range [IQR]), box and whisker plots (25^th^ and 75^th^ percentiles), means ± SD, or numbers (%), as appropriate. Unless otherwise stated, comparisons were performed between the traditional and ERAS groups. Statistical evaluations were performed using two-way analysis of variance, Wilcoxon’s signed-ranks test for continuous outcomes, and Fisher’s exact test for binary outcomes. All statistical analyses were performed using the JMP11 software program (SAS institute Japan LTD., Tokyo, Japan). P < 0.05 was considered statistically significant.

## Results

Of the 352 consecutive colorectal cancer resections performed at Tokyo Metropolitan Bokutoh Hospital that met the inclusion criteria, 95 were in the traditional group and 257 were in the ERAS group. Patient characteristics are shown in Table 2. The two groups were similar in sex, age, body mass index, ASA performance status, and tumor location (all P > 0.05). The surgical approach varied between the two groups, with all operations being open surgeries in the traditional group, and one-fifth performed laparoscopically in the ERAS group (P < 0.05). This was due to the adoption of laparoscopic surgery for colorectal cancer during the study period, starting in July 2011. There was no significant difference in the distribution of colorectal cancer stages. The number of dissected lymph nodes was 32.9 ± 20.2 in the traditional group and 33.6 ± 19.7 in the ERAS group, with no significant difference. These results suggest that sufficient lymph node dissection was performed in both groups. Overall compliance with ERAS protocols was good. With regard to postoperative feeding, none of the patients in the traditional group ate rice at postoperative day (POD) 3, whereas 68 % of patients in the ERAS group ate rice at POD3.

**Table 2:** Patient characteristics

	Traditional	ERAS
	2009.07-2010.06	2010.07-2012.11
	n = 95	n = 257
Sex (male/female)	65/30	165/92
Age (years)	69.1 ± 9.0	68.8 ± 11.5
Body mass index (kg/m^2^)	22.3 ± 3.1	22.0 ± 3.5
ASA (1/2/3)	3/78/14	3/211/43
Tumor location (colon/rectum)	59/36	168/89
Surgical approach (open/laparoscopic)	95/0	203/54*
Number of retrieved lymph nodes	32.9 ± 20.2	33.6 ± 19.7
Stage (0/I/II/III/IV)	1/7/27/39/21	6/37/90/85/39

*p < 0.05

Intraoperative outcomes are summarized in Table 3. Operative time and blood loss did not significantly differ between the two groups. The volume of intraoperative infusion was significantly lower in the ERAS group (P < 0.05).

**Table 3:** Intraoperative outcomes

	Traditional	ERAS
Operative time (min)	222.3 ± 106.2	205.6 ± 70.5
Blood loss (gram)	639.0 ± 744.8	516.6 ± 740.2
Volume of intraoperative infusion (ml)	3218.2 ± 2100.3	2232.7 ± 1168.6*

*p < 0.05

**Fig. 1:**
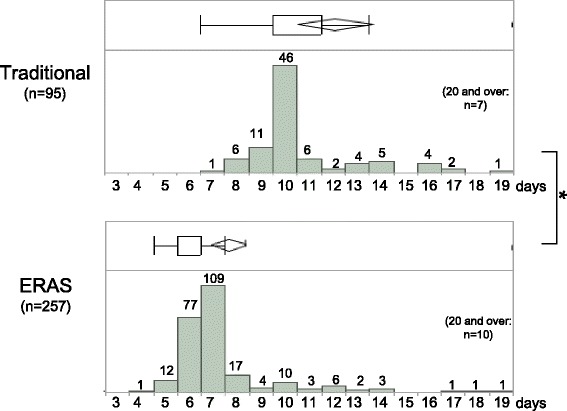
Postoperative length of hospital stay (LOS) is shown as a histogram and box and whisker plots (25^th^, 75^th^ percentiles) for both traditional and ERAS groups. Median LOS is indicated by horizontal bars, and mean LOS by rhombuses. Horizontal boundaries of the boxes represent the first and third quartiles. *P < 0.05

Outcomes regarding LOS are shown in Fig. 1. The median (IQR) postoperative LOS was 10 (10–12) days in the traditional group and seven (6–7) days in the ERAS group, showing a three-day reduction in the ERAS group (P < 0.05). In histograms and box and whisker plots (25^th^, 75^th^ percentiles) (Fig. 1), horizontal bars correspond to median LOS (10 in the traditional group and seven in the ERAS group), and rhombuses correspond to mean LOS (12.7 and 8.2, respectively). The first and third quartiles (10 and 12, 6 and 7, respectively) are represented by horizontal boundaries of the boxes. There was a dramatic increase in the proportion of patients who were discharged within one week, from 1 % in the traditional group to 77 % in the ERAS group.

Since laparoscopic surgery, which is generally considered less invasive, was performed only in the ERAS group, we compared the LOS between patients who had undergone laparoscopic surgery and those who had undergone open surgery within the ERAS group, in order to account for differences due solely to the type of surgical approach. A significant difference was found in the median LOS between laparoscopic surgery (six days) and open surgery (seven days) (P < 0.05).

Postoperative outcomes are summarized in Table 4. Overall, 9.5 % of patients in the traditional group and 9.3 % in the ERAS group had one or more postoperative complications. Rates of anastomotic leakage, postoperative ileus, pneumonia, cardiac disorders, and overall complications did not significantly differ between the two groups. While there was no mortality in the traditional group, one patient in the ERAS group died during the postoperative course due to myocardial infarction. The rate of reoperation within 30 days was 2.1 % in the traditional group and 1.6 % in the ERAS group, with no significant difference. There was also no significant difference in 30-day readmission rates between traditional and ERAS groups (8.3 % and 6.6 %, respectively).

**Table 4:** Postoperative outcomes

	Traditional	ERAS
Postoperative complications	9 (9.5 %)	24 (9.3 %)
anastomotic leakage	2 (2.1 %)	5 (1.9 %)
ileus	3 (3.1 %)	9 (3.5 %)
pneumonia	1 (1.0 %)	4 (1.6 %)
cardiac disorder	1 (1.0 %)	1 (0.4 %)
other	2 (2.1 %)	5 (1.9 %)
Readmission within 30 days	8 (8.3 %)	17 (6.6 %)
Reoperation within 30 days	2 (2.1 %)	4 (1.6 %)
Mortality	0 (0 %)	1 (0.4 %)

## Discussion

Japan has one of the best outcomes in the world for colorectal cancer treatment, and has its own traditions of perioperative care along with a unique insurance system. Whether or not the introduction of ERAS protocols benefits the Japanese population has been unclear. In the present study, we demonstrated that ERAS protocols for colorectal surgery can be applied and integrated effectively in a general hospital setting in Japan. In other words, ERAS protocols can help achieve faster patient recovery without increasing postoperative morbidity or readmission rates in Japan.

Although operative time and blood loss did not differ between the two groups, the volume of intraoperative infusion was significantly lower in the ERAS group. This might be explained by ERAS recommendations to avoid dehydration, i.e., the avoidance of sodium/fluid overload and no preoperative fasting. All intraoperative infusions were exclusively performed by anesthesiologists, suggesting the importance of the multimodal approach of ERAS.

In Japan, traditional perioperative care for gastrointestinal surgery includes “*nil per os* for several days.” ERAS protocols were introduced in Japan around 2010, and have since spread steadily to clinical institutions across the country. In 2012, the Japanese Society of Anesthesiologists established guidelines for preoperative fasting and oral rehydration therapy. Today, many hospitals in Japan perform preoperative oral rehydration therapy prior to gastrointestinal surgery [9].

In our study, different surgical approaches were used in the traditional and ERAS groups, with all operations being open surgeries in the traditional group, and approximately one-fifth of operations performed laparoscopically in the ERAS group. Laparoscopic colorectal resection has been shown to reduce postoperative pain, is generally considered less invasive, and is expected to result in a shorter LOS compared to open surgery. The JCOG0404 study, a randomized controlled trial in Japan, evaluated laparoscopic versus open colorectal surgery with D3 dissection, and reported that the LOS was 10 days following laparoscopic surgery, as opposed to 11 following open surgery [17]. The COLOR II study, a randomized phase III trial that compared laparoscopic surgery and open surgery for rectal cancer in eight European countries, had similar findings: postoperative LOS was eight days for laparoscopic surgery and nine for open surgery [18]. In both studies, the difference in LOS between laparoscopic surgery and open surgery was significant, albeit by only one day [17, 18]. Our results were consistent in that the median LOS was six days in patients undergoing laparoscopic surgery, as opposed to seven days in those undergoing open surgery, in the ERAS group. These findings suggest that the reduction in LOS by three days, as observed in the ERAS group compared to the traditional group, was not due solely to laparoscopic surgery.

Both ERAS protocols and laparoscopic surgery represent recent major changes in colorectal perioperative care that have led to improved clinical outcomes following colorectal cancer surgery. However, questions have been raised recently regarding whether or not the benefits of laparoscopy still exist when open surgery is optimized due to ERAS. In the United Kingdom, a multicenter randomized controlled trial of conventional versus laparoscopic surgery for colorectal cancer with an ERAS program showed that, while patients treated by experienced surgeons according to ERAS had similar physical fatigue and patient-reported outcomes in both groups, laparoscopic surgery significantly reduced the LOS (median: laparoscopy, five days vs. open, seven days) [19]. These results support the observation that ERAS benefits colorectal cancer patients regardless of surgical approach.

The major limitation of this study is that it was a historical control study. As mentioned above, due to Japan’s unique governmental health insurance system, some patients may prefer to stay in the hospital longer. As such, LOS may not necessarily reflect the patient’s medical and physical status. However, in recent years, hospitals have been under increasing pressure to reduce LOS, as the government sees that this could contribute to a reduction in national medical costs. In this context, it is clear that the main purpose of introducing ERAS protocols is the reduction of postoperative LOS. The actual LOS might have been influenced by patients being pressured by surgeons or medical staff to hasten discharge.

## Conclusions

In summary, although Japan follows distinct protocols to treat colorectal cancer as compared with European countries and the United States, and has a unique health insurance system that allows patients to delay discharge after surgery, the present study demonstrated the effectiveness of ERAS protocols to speed up patient recovery in Japan, without increasing postoperative morbidity or readmission rates. Thus, colorectal ERAS protocols should be applied and integrated in general hospital settings in Japan.
